# Oxytocinergic Feedback Circuitries: An Anatomical Basis for Neuromodulation of Social Behaviors

**DOI:** 10.3389/fncir.2021.688234

**Published:** 2021-06-14

**Authors:** Arthur Lefevre, Diego Benusiglio, Yan Tang, Quirin Krabichler, Alexandre Charlet, Valery Grinevich

**Affiliations:** ^1^Department of Neuropeptide Research in Psychiatry, Central Institute of Mental Health, Medical Faculty Mannheim, University of Heidelberg, Mannheim, Germany; ^2^European Molecular Biology Laboratory (EMBL), Epigenetics and Neurobiology Unit, Monterotondo, Italy; ^3^Neuroscience and Behaviour Laboratory, Istituto Italiano di Tecnologia, Rome, Italy; ^4^Department of Biomedical Sciences, Faculty of Biology and Medicine, University of Lausanne, Lausanne, Switzerland; ^5^Centre National de la Recherche Scientifique (CNRS) and University of Strasbourg, Institute of Cellular and Integrative Neurosciences, Strasbourg, France

**Keywords:** oxytocin, oxytocin receptor (OTR), social brain, anatomy, loops

## Abstract

Oxytocin (OT) is a neuropeptide produced by hypothalamic neurons and is known to modulate social behavior among other functions. Several experiments have shown that OT modulates neuronal activity in many brain areas, including sensory cortices. OT neurons thus project axons to various cortical and subcortical structures and activate neuronal subpopulations to increase the signal-to-noise ratio, and in turn, increases the saliency of social stimuli. Less is known about the origin of inputs to OT neurons, but recent studies show that cells projecting to OT neurons are often located in regions where the OT receptor (OTR) is expressed. Thus, we propose the existence of reciprocal connectivity between OT neurons and extrahypothalamic OTR neurons to tune OT neuron activity depending on the behavioral context. Furthermore, the latest studies have shown that OTR-expressing neurons located in social brain regions also project to other social brain regions containing OTR-expressing neurons. We hypothesize that OTR-expressing neurons across the brain constitute a common network coordinated by OT.

## Introduction

Oxytocin (OT) is a neuropeptide mainly synthesized in the paraventricular (PVN), supraoptic (SON), and accessory nuclei of mammalian hypothalamus and is present, with some minor molecular variations, in all vertebrates (Knobloch and Grinevich, 2014; Banerjee et al., 2016). This peptide has two general ways of action: first, *via* projections to the posterior pituitary, it is secreted into the bloodstream as a hormone controlling various physiological processes, such as parturition, lactation, energetic metabolism, cardiovascular function, bone homeostasis, and muscle maintenance (Neumann et al., 1993; Gutkowska and Jankowski, 2012; Chaves et al., 2013; Kasahara et al., 2013; Elabd et al., 2014; Poisbeau et al., 2017; Sun et al., 2019). Secondly, OT acts in the brain as a non-canonical neurotransmitter or neuromodulator, regulating a number of behaviors ranging from pain to social behaviors (Macdonald and Feifel, 2014; Bowen and Neumann, 2017; Grinevich and Stoop, 2018; Lawson et al., 2019).

In this mini-review, we will primarily focus on brain OTergic circuits, which modulate social behavior. The current leading hypothesis to explain OT effects on social behavior is that the neuropeptide selectively increases the saliency of socially relevant stimuli in areas enriched with OTR-expressing neurons (Shamay-Tsoory and Abu-Akel, 2015; Marlin and Froemke, 2016). This hypothesis is mainly based on results obtained in studies of the auditory and olfactory centers, where OT modulation acts on the excitation/inhibition balance of sensory circuits to increase the signal to noise ratio in favor of social stimuli and by this mechanism filter out less relevant stimuli (Marlin et al., 2015; Oettl et al., 2016; Linster and Kelsch, 2019). Although it is becoming clearer how OT may affect sensory systems, the mechanisms underlying targeted axonal release of OT in the socially relevant brain regions remain elusive. While an extensive series of tracing experiments were performed in the 1970s and 1980s (Sawchenko and Swanson, 1983), only a few recent studies have described the inputs and outputs of OT neurons with modern neuroanatomical techniques (Grinevich and Stoop, 2018; Son et al., 2020; Tang et al., 2020; Zhang et al., 2020). Thus, here we will first review outputs of OT neurons and their effects with an emphasis on cortical sensory regions. We will then synthesize recent reports on inputs to OT neurons, suggesting the existence of functional feedback loops between OT neurons and OTR-containing regions. Finally, we will propose a hypothesis that brain regions containing OTR form interconnected networks to regulate various forms of complex social behaviors.

## Brain-Wide OT Modulation

In addition to the well-described somatodendritic release of OT, which takes place within the hypothalamic nuclei, the PVN and SON, specifically during lactation (Landgraf and Neumann, 2004; Ludwig and Leng, 2006; Tobin et al., 2014), OT neurons project distant axons throughout most of the forebrain and parts of the brain stem (Knobloch et al., 2012; Zhang et al., 2020), releasing a small number of large dense-core vesicles containing OT within a target region in non-synaptic fashion (Chini et al., 2017). The distribution of OT axonal terminals largely overlaps with OTR in target brain areas (Tribollet et al., 1988, 1991; Campbell et al., 2009; Grinevich et al., 2016; Mitre et al., 2016).

In various subcortical regions innervated by OT axons, the neuropeptide release is known to attenuate anxiety, fear, and physiological stress responses. Specifically, OT modulation of neural circuits in the central amygdala reduces contextual fear response (Knobloch et al., 2012; Hasan et al., 2019) and anxiety (Wahis et al., 2021), and in the lateral septum (LS) prevents social fear during lactation (Menon et al., 2018) as well as decreases aggression of female virgins (Oliveira et al., 2020). OT axons also reach the paraventricular nucleus of the thalamus (PVT) promoting maternal behavior (Knobloch et al., 2012; Cilz et al., 2018; Watarai et al., 2020). Furthermore, the OT system interacts with the serotonergic system by projecting to the raphe nucleus, promoting socially rewarding behaviors (Dölen et al., 2013). Up to date, the global OT projections through the entire brain have been mapped (Knobloch et al., 2012; Son et al., 2020), but the outputs of individual OT neurons or their subpopulations remain to be explored. So far, subpopulations of OT neurons in rodents have been shown to selectively target only a few distinct extrahypothalamic regions (Menon et al., 2018; Ferretti et al., 2019; Hasan et al., 2019), but do not uniformly send axonal collaterals to all OTR-expressing regions. A recent study mapping individual OT neuron projections by Levkowitz’s group in fish confirmed these results (Wircer et al., 2017). Thus, it is likely that the OT system is composed of anatomically and functionally distinct clusters (Althammer and Grinevich, 2017), which specifically modulate OT-sensitive brain regions, controlling distinct forms of behaviors (Menon et al., 2018; Oliveira et al., 2020). Elucidating the functional organization of these different ensembles of OT neurons in mammals including humans will be an important challenge for future research.

## Long-Range Oxytocin Modulation of Sensory Cortical Circuits

In the cortex, the projections from OT neurons reach several sensory cortical areas, such as primary auditory, olfactory, and somatosensory cortices, where OT regulates the processing of sensory stimuli *via* enhancement of social cues’ saliency (Marlin and Froemke, 2016; Mitre et al., 2016). This principle is exemplified by the study of Oettl et al. who showed that endogenous release of OT in the anterior olfactory nucleus (AON) of the olfactory cortex increases its excitatory drive and activates its top-down projections to granule cells in the olfactory bulb, enhancing the signal-to-noise ratio of social odor responses. In addition, the authors showed that optogenetically evoked release of OT from axons residing in the AON stimulates olfactory exploration and social recognition, while ablation of OTR in this cortex resulted in a “social amnesia” (Oettl et al., 2016).

Another notable example is the role of OT signaling in the primary auditory cortex (A1) that enables the initiation of pup retrieval, a specific maternal care behavior in female mice (Noirot, 1972). Mouse pups isolated from the nest emit ultrasonic distress calls that experienced mothers (called “dams”) recognize and use to find and retrieve them to the nest. Conversely, virgin inexperienced females do not retrieve pups, but they start retrieving pups after being co-housed with dam and pups (Ehret et al., 1987). Interestingly, female mice that learned to retrieve pups show a higher neural response in the auditory cortex to pup distress calls than naive ones (Liu et al., 2006). Froemke’s group reported that OT is crucial to drive the cortical plasticity occurring in the auditory cortex of mice which initiated pup retrieval behavior (Marlin et al., 2015; Schiavo et al., 2020). The recruitment of OTR neurons in the left auditory cortex increases the signal-to-noise ratio of pup calls responses of principal neurons, enabling efficient pup retrieval by their mothers as well as experienced virgins trained by lactating dams. However, the question whether OT influences plasticity related to other auditory learning tasks needs further investigation.

OT- and experience-induced cortical plasticity are not exclusive to auditory processing. Rather, this seems to be a generalized principle occurring during critical physiological transitions—such as motherhood (Brecht et al., 2018; Valtcheva and Froemke, 2018). Intriguingly, the area representing the nipples and areolae in the somatosensory barrel cortex (S1) is largely expanded (a two-fold increase) in lactating rats, and this is induced by somatosensory stimulations in the form of suckling, artificial suction, or nipple rubbing (Rosselet et al., 2006). Interestingly, the S1 barrel field receives OT projections from the PVN (Grinevich et al., 2016) and expresses OTR (Newmaster et al., 2020). Thus, it is tempting to propose that OT may facilitate nursing-induced cortical plasticity because the neuropeptide concentration in S1 is gradually increased after prolonged sensory stimulation of the nipples (Zheng et al., 2014). Similarly, sensory deprivation in mice *via* whisker trimming or dark rearing after birth leads to reduced synaptic transmission in somatosensory and visual cortices, respectively, as well as to abolished OT synthesis, release, and overall OT neuron activity (Zheng et al., 2014), supporting the involvement of OT in the condition-dependent cortical plastic changes.

## Do OT Neurons Receive Feedback Projections from Their Targets?

Although OT projections have been extensively studied in various brain regions, little is known on which sensory modalities trigger the activation of these neurons. The pioneering works performed around the end of the last century employing conventional antero- and retrograde tracers showed a number of extrahypothalamic regions projecting to the PVN and SON without discrimination of cell types within these nuclei (Sawchenko and Swanson, 1983; Iovino et al., 2016) followed by verification of the synapses onto OT neurons by electrophysiology (Hatton and Yang, 1989; Leng et al., 1999) or electron microscopy (Oldfield et al., 1985; Cservenák et al., 2016).

**Figure 1:**
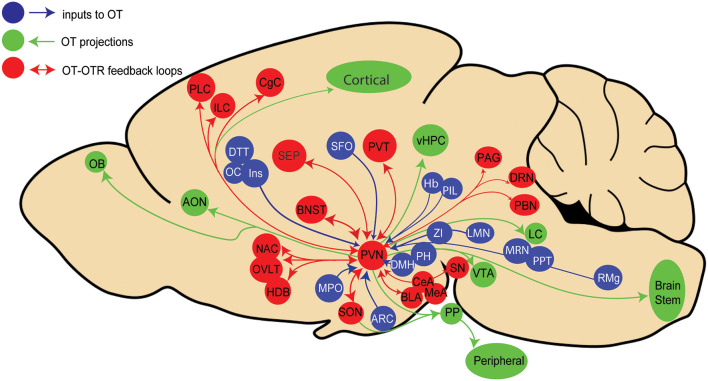
Schema representing known paraventricular (PVN) oxytocin (OT) neurons inputs (blue), outputs (green), and areas providing both inputs to and receiving projections from PVN OT neurons in rats. Brain areas legend: AON, Accessory olfactory nucleus; Amygdala: Amy (central, basolateral and medial amygdala: CeA,BLA, MeA), ARC, Arcuate hypothalamic nucleus; BNST, Bed nucleus of stria terminalis; CgC, Cingulate cortex; Cl, Claustrum; DRN, Dorsal raphe nucleus; DTT, Dorsaltenia tecta; DMH, Dorsomedial hypothalamic area; Hb, Habenular nucleus; HDB, Horizontal limb of diagonal band nucleus; ILC, Infralimbic cortex; Ins, Insular cortex; LH, Lateral hypothalamic area; LMN, Lateral lemniscus nucleus; SEP, Lateral septal nucleus; LC, Locus Coeruleus; MMB, Mammillary body; MPO, Medial preoptic area; MRN, Median raphe nucleus; NAC, Nucleus accumbens; OB, Olfactory bulb; OC, Orbital cortex; PBN, Parabrachial nucleus; PVT, Paraventricular thalamus; PPT, Pedunculopontine tegmental nucleus; PAG, Periaqueductal gray area; PH, Posterior hypothalamic nucleus; PIL, Posterior intralaminar thalamus; PLC, Prelimbic cortex; PP, Posterior Pituitary; RMg, Raphe magnus nucleus; SFO, Subfornical organ; SN, Substancia nigra; OVLT, Vascular organ of lamina terminalis: vHPC, ventral hippocampus; VTA, Ventral tegme ZI, Zona incerta.

Recent advances in viral vector-based technology have allowed us to more precisely trace the origin of synaptic inputs to a genetically defined cell population (Wickersham et al., 2007). Using cell-type-specific OT promoter inserted into viral vectors in rats, we mapped neurons that synapse onto OT neurons in the PVN (Tang et al., 2020) and we found that 22 extrahypothalamic regions terminate on OT neurons. Among the previously known inputs (Sawchenko and Swanson, 1983) we identified new regions projecting to OT neurons, such as the infralimbic and insular cortices (Figure 1).

Similar results were recently obtained in mice (Son et al., 2020), although a number of input structures were distinct from those identified in rats. The most important discrepancy in mice is the absence of input to OT neurons from cortical areas, such as the prelimbic and infralimbic, cingulate, orbital, and insular cortices as was shown in rats (Tang et al., 2020). This may reflect a joint evolution of increasingly complex social behaviors and their neuronal underpinnings in rats compared to mice. We also found a large number of input cells in the subfornical organ, a known source of innervation to OT neurons (Anderson et al., 2001), that was not reported by Son et al. (2020), probably because this structure was not taken into account during the analysis.

While in both rodent species primary sensory cortices do not project to OT neurons, secondary sensory areas, such as the posterior and auditory thalamic nuclei do (Dobolyi et al., 2018; Valtcheva et al., 2021). This suggests that sensory information in rodents is conveyed to the OT system *via* secondary pathways, which could explain the activation of OT neurons by diverse sensory channels involved in social communication such as tactile (Tang et al., 2020) and auditory stimuli (Valtcheva et al., 2021), fear (Hasan et al., 2019), pain (Eliava et al., 2016), and reproductive and parental behaviors (Scott et al., 2015). However, we still do not know whether OT neurons and/or their subpopulations can be categorized based on their specific inputs (as well as outputs; Hasan et al., 2019).

Nevertheless, these recent results unveil an aspect, which was not previously considered: most regions receiving inputs from OT neurons, and/or expressing OTR seems to project back to OT neurons, thus potentially forming reciprocal connections between OT neurons and their target neurons expressing OTRs (Figure 1). However, it is as yet unclear whether these feedback connections are emanating from OTR neurons or non-OTR neurons. Thus, the behavioral role of such feedback loops to OT neurons remains to be elucidated.

## Do OTR Neurons in Distant Brain Regions Communicate to Each Other?

Although OTR neurons are present in the vast majority of forebrain regions, the anatomical and functional connectivity between them has only been explored by a few studies. While the dominating view is that OTR neurons generally represent GABAergic local interneurons (Marlin and Froemke, 2016), it is now known that principal glutamatergic neurons, as well as astrocytes, are also capable to express OTR (Mitre et al., 2016; Tan et al., 2019; Wahis et al., 2021).

Using OTR-Cre knock-in mice in combination with a virus expressing GFP in a Cre dependent manner, a first study showed that VTA OTR neurons project to the medial prefrontal cortex (mPFC), nucleus accumbens (NAcc), amygdala (Amy), and lateral habenula (LHb; Peris et al., 2016). On the same line, another study, using a similar strategy revealed that OTR neurons in the mPFC project to the bed nucleus of stria terminalis (BNST), the NAcc, and Amy (Tan et al., 2019). Employing a different approach, Dölen and colleagues injected the retrograde monotranssynaptic rabies virus expressing tdTomato in the NAcc of OTR-Venus mice and found that NAcc (itself containing OTR cells) received direct inputs from OTR cells in some distant areas such as accessory olfactory nuclei (AON), mPFC, Amy, paraventricular thalamic nucleus (PVT), CA1, dorsal raphe nucleus (DRN), and ventral tegmental area (VTA; Dölen et al., 2013). Finally, similar findings have been obtained in novel OTR-Cre knock-in prairie voles that were recently generated by CRISPR/Cas9 technology (Horie et al., 2020). The authors showed that the prairie vole’s NAcc receives direct inputs from OTR neurons of various areas, such as the AON, mPFC, Amy, cingulate cortex, PVT, and insula. Together, these findings suggest that the existence of long-range projecting OTR neurons is not exceptional, but rather a common feature of the brain OT system.

**Figure 2:**
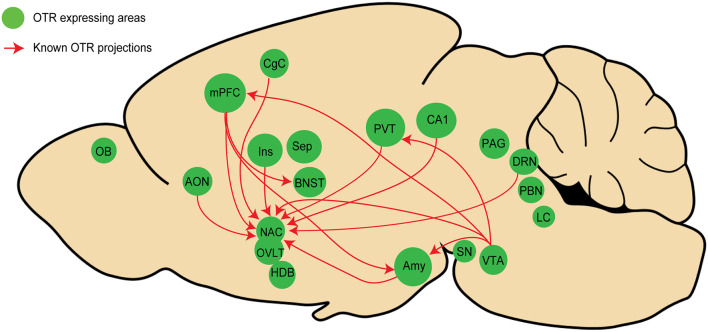
Schema representing the known inter areas OT receptor (OTR) network in rodents. Regions in green contain OTR neurons, red arrows represent OTR projections in other regions containing OTR neurons. Note that only four studies (see text) have analyzed OTR projections, mostly focusing on the NAcc, indicating that numerous OTR connections have not been yet discovered. See Figure 1 legends for abbreviations.

When summarizing all known connections between areas containing OTR neurons (as we have endeavored to do in Figure 2), it is striking that most of them are located in regions that are part of the “social brain,” a network of areas regulating social behavior (Olsson et al., 2020). It is important to note that these networks are not necessarily involving OTR neurons at the postsynaptic level, meaning that as far as we know, OTR neurons projecting to a distant area could contact other OTR or non-OTR neurons. Although the role of a potential “OTR network” remains to be determined, we hypothesize that such a network is important for the regulation of social behaviors. Experimental evidence indicates that the stimulation of fibers originating from OTR neurons in the mPFC and terminating in the basolateral amygdala (BLA) disrupts social recognition in mice (Tan et al., 2019). Thus, OTR to OTR neurons could represent an anatomical substrate essential for synchronization of activity in this network, allowing OT to produce a coherent (e.g., “whole brain”) response. This hypothesis should be addressed together with other key questions regarding the functional organization of the OT system, such as whether OT is released selectively or simultaneously in all these brain areas (i); to what degree OT-sensitive circuitries connect to each other, at least in the “social brain” (ii); and how activation of subpopulations of OT neurons upon distinct sensory modalities is transmitted to various OTR expressing brain regions (iii).

Several recent technological developments render possible the exploration of these questions. For instance, using pseudotype rabies infecting cells in a cre dependent manner combined with transgenic OTR-Cre animals could bring evidence that OTR neurons in one region are innervated by OTR neurons from another region. Another useful tool is the generation of non-toxic pseudotype rabies (Ciabatti et al., 2017; Chatterjee et al., 2018) that will allow functional interrogation of defined inputs to OT neurons. Finally, a new type of sensor (Wang et al., 2018) can be used to track OT concentration in multiple areas of the brain, allowing one to investigate question (i).

## Conclusions/Perspectives

In this review, we proposed two novel features of OT signaling in the brain. First, we showed that OT neurons received reciprocal input from the OT-sensitive structures they are innervating. Although the functional significance of such potential feedback projections is not clear, it is tempting to postulate that OTR neurons of distant brain regions dynamically tune “positive” vs. “negative” balance of OT neurons to initiate, accelerate, or slow down behavioral responses. Secondly, we hypothesized that OTR neurons in spatially distant brain regions are communicating with each other to further control social behaviors elicited by an initial OT release. An important note is that all the studies reviewed here have been performed in rodents, and thus whether the proposed circuits might also exist in other mammals is unknown. Finally, we would like to point out that OT is the most studied neuropeptide at the moment, but that the mechanisms proposed here can be expanded towards other less renown neuropeptides, which are also important neuromodulators of social behaviors (Lefevre et al., 2018).

## Author Contributions

All authors discussed and elaborated the ideas, wrote the first draft and corrected it until the final version was obtained. All authors contributed to the article and approved the submitted version.

## Conflict of Interest

The authors declare that the research was conducted in the absence of any commercial or financial relationships that could be construed as a potential conflict of interest.
